# Poly[dimethyl­ammonium [aquadi-μ_2_-oxalato-dysprosate(III)] trihydrate]

**DOI:** 10.1107/S1600536810026140

**Published:** 2010-07-07

**Authors:** Su-Fang Ye, Hong Lin

**Affiliations:** a Jinhua Professional Technical College, Jinhua, Zhejiang 321007, People’s Republic of China

## Abstract

The title compound, {(C_2_H_8_N)[Dy(C_2_O_4_)_2_(H_2_O)]·3H_2_O}_*n*_, was obtained as an unexpected product under hydro­thermal conditions. The Dy^III^ atom is chelated by four oxalate anions, two of which are situated on two different centres of inversion. The distorted tricapped trigonal-prismatic coordination sphere of the Dy^III^ atom is completed by a water mol­ecule. The bridging mode of the anions results in the formation of a three-dimensional network with cavities where the ammonium cations and the uncoordinated water mol­ecules reside. The structure is stabilized by numerous N—H⋯O and O—H⋯O hydrogen-bonding inter­actions.

## Related literature

For decomposition mechanisms of organic ligands resulting in the formation of oxalates, see: Ghosh *et al.* (2004 ▶); Zhong *et al.*, (2008 ▶). For other Dy^III^ oxalate compounds, see: Hansson (1973 ▶); Kahwa *et al.* (1984 ▶); Ollendorff *et al.* (1969 ▶). The structure of the isotypic Eu^III^ compound was reported by Yang *et al.* (2005 ▶).
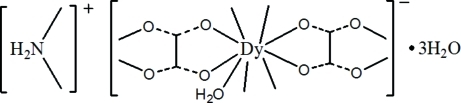


## Experimental

### 

#### Crystal data

(C_2_H_8_N)[Dy(C_2_O_4_)_2_(H_2_O)]·3H_2_O
*M* *_r_* = 456.70
Monoclinic, 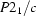

*a* = 9.6239 (2) Å
*b* = 11.6030 (2) Å
*c* = 14.3050 (2) Å
β = 122.463 (1)°
*V* = 1347.77 (4) Å^3^
*Z* = 4
Mo *K*α radiation
μ = 5.61 mm^−1^
*T* = 296 K
0.19 × 0.15 × 0.04 mm

#### Data collection

Bruker APEXII area-detector diffractometer
Absorption correction: multi-scan (*SADABS*; Sheldrick, 1997 ▶) *T*_min_ = 0.374, *T*_max_ = 0.790
20173 measured reflections
3108 independent reflections
2810 reflections with *I* > 2σ(*I*)
*R*_int_ = 0.032

#### Refinement

*R*[*F* ^2^ > 2σ(*F* ^2^)] = 0.019
*wR*(*F* ^2^) = 0.047
*S* = 1.06
3108 reflections
211 parameters
12 restraints
H atoms treated by a mixture of independent and constrained refinement
Δρ_max_ = 0.84 e Å^−3^
Δρ_min_ = −0.91 e Å^−3^

### 

Data collection: *APEX2* (Bruker, 2006 ▶); cell refinement: *SAINT* (Bruker, 2006 ▶); data reduction: *SAINT*; program(s) used to solve structure: *SHELXS97* (Sheldrick, 2008 ▶); program(s) used to refine structure: *SHELXL97* (Sheldrick, 2008 ▶); molecular graphics: *SHELXTL* (Sheldrick, 2008 ▶) and *DIAMOND* (Crystal Impact, 2008 ▶); software used to prepare material for publication: *SHELXTL*.

## Supplementary Material

Crystal structure: contains datablocks I, global. DOI: 10.1107/S1600536810026140/wm2367sup1.cif

Structure factors: contains datablocks I. DOI: 10.1107/S1600536810026140/wm2367Isup2.hkl

Additional supplementary materials:  crystallographic information; 3D view; checkCIF report

## Figures and Tables

**Table 1:** Selected bond lengths (Å)

Dy1—O3^i^	2.3846 (17)
Dy1—O2	2.3883 (19)
Dy1—O5	2.390 (2)
Dy1—O6	2.427 (2)
Dy1—O1	2.4335 (18)
Dy1—O4^i^	2.4386 (19)
Dy1—O8	2.445 (2)
Dy1—O1*W*	2.451 (2)
Dy1—O7	2.464 (2)

Symmetry code: (i) 
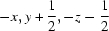
.

**Table 2:** Hydrogen-bond geometry (Å, °)

*D*—H⋯*A*	*D*—H	H⋯*A*	*D*⋯*A*	*D*—H⋯*A*
N1—H1*A*⋯O8	0.89 (4)	2.00 (4)	2.866 (4)	163 (4)
N1—H1*A*⋯O1*W*	0.89 (4)	2.52 (4)	3.090 (4)	122 (3)
O1*W*—H1*WA*⋯O3*W*	0.84 (4)	2.03 (2)	2.857 (4)	173 (3)
O4*W*—H4*WB*⋯O2*W*	0.82 (4)	2.06 (3)	2.767 (4)	144 (5)
N1—H1*B*⋯O4*W*^ii^	0.87 (4)	1.91 (4)	2.759 (5)	165 (4)
O1*W*—H1*WB*⋯O3*W*^iii^	0.84 (4)	1.92 (2)	2.744 (3)	167 (3)
O2*W*—H2*WB*⋯O3^iv^	0.85 (4)	2.06 (2)	2.876 (4)	161 (5)
O3*W*—H3*WA*⋯O7^v^	0.85 (4)	2.24 (3)	2.959 (3)	145 (4)
O3*W*—H3*WA*⋯O6^vi^	0.83 (4)	2.34 (2)	3.110 (3)	156 (4)
O3*W*—H3*WB*⋯O4*W*^vii^	0.80 (5)	2.42 (3)	2.959 (5)	125 (4)
O3*W*—H3*WB*⋯O2*W*^vii^	0.80 (5)	2.49 (2)	3.256 (5)	160 (4)
O4*W*—H4*WA*⋯O4^viii^	0.84 (4)	2.10 (3)	2.837 (4)	147 (5)

Symmetry codes: (ii) 
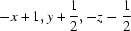
; (iii) 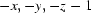; (iv) 
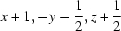
; (v) 
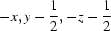
; (vi) 
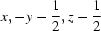
; (vii) 
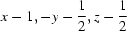
; (viii) 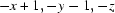.

**Fig. 1.**
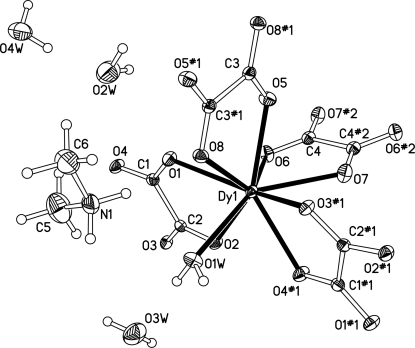
Perspective view of the title compound, showing the atom-numbering scheme. Displacement ellipsoids are drawn at the 30% probability level. Symmetry codes: (1) -x, y+0.5, -z-0.5; (2) -x, -y, -z.

**Fig. 2.**
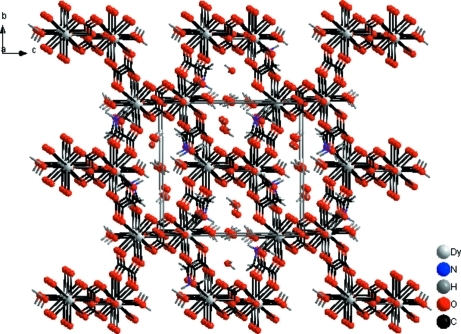
A view of the three-dimensional polymeric network of the title compound.

## supplementary crystallographic information

### Comment

Multidentate organic ligands are usually engaged in the construction of complexes, among which oxalate is one of the simplest imaginable connectors potentially able to bridge metal ions in a bidentate chelating manner. Some dysprosium(III) oxalate compounds, such as K_8_[Dy_2_(C_2_O_4_)_7_]^.^14H_2_O (Kahwa *et al.*, 1984) and [Dy_2_(C_2_O_4_)(H_2_O)_6_]^.^4H_2_O (Ollendorff *et al.*, 1969; Hansson 1973) have been reported. Oxalates usually represent one of the main end-products of the degradation of some organic ligands, under both oxidative and nonoxidative conditions (Ghosh *et al.*, 2004). For example, decomposition of pyridine-2,4,6-tricarboxylic acid into oxalate has been observed in the presence of cadmium(II) compounds (Zhong *et al.*, 2008). Herein, we report a new three-dimensional oxalate structure, (C_2_H_8_N)[Dy(C_2_O_4_)_2_(H_2_O)].3H_2_O.

A view on the molecular structure of the title compound, (I), which is isotypic with its Eu(III) analogue (Yang *et al.*, 2005), is presented in Fig. 1. The central Dy^III^ atom displays a distorted tricapped trigonal-prismatic coordination by four oxalate anions and one water molecule. Each Dy^III^ atom is connected to four adjacent Dy^III^ centres through the oxalate bridges resulting in a three-dimensional polymeric network as depicted in Fig. 2. The Dy—Dy separations are 6.2135 (2), 6.2742 (2) and 6.3164 (3) Å, respectively. The cations and solvent water molecules occupy the cavities of the network and are involved in hydrogen-bonding with each other and with the network. This gives rise to a tightly held network structure.

### Experimental

A mixture of 2-carboxymethylsulfanyl nicotinic acid (0.086 g, 0.40 mmol), Dy_2_O_3_ (0.093 g, 0.25 mmol) in DMF (5 ml)/H_2_O (15 ml) was placed in a 25 ml Teflon-lined stainless steel reactor and heated at 433 K for 72 h, and then cooled to room temperature over 3 days. Colourless crystals were obtained in approximate 30% yield.

### Refinement

The C-bound and ammonium H-atoms were positioned geometrically and included in the refinement using a riding model [C—H 0.96 Å and N—H 0.87, 0.89 *U*_iso_(H) = 1.2*U*_eq_(C)]. The water H atoms were located from difference maps, and their positions were refined with O—H distances fixed at 0.85 (5) Å with *U*_iso_(H) = 1.2*U*_eq_(O).

### Crystal data

**Table:**

(C_2_H_8_N)[Dy(C_2_O_4_)_2_(H_2_O)]·3H_2_O	*F*(000) = 884
*M**_r_* = 456.70	*D*_x_ = 2.251 Mg m^−^^3^
Monoclinic, *P*2_1_/*c*	Mo *K*α radiation, λ = 0.71073 Å
Hall symbol: -P 2ybc	Cell parameters from 9337 reflections
*a* = 9.6239 (2) Å	θ = 2.4–27.6°
*b* = 11.6030 (2) Å	µ = 5.61 mm^−^^1^
*c* = 14.3050 (2) Å	*T* = 296 K
β = 122.463 (1)°	Block, colourless
*V* = 1347.77 (4) Å^3^	0.19 × 0.15 × 0.04 mm
*Z* = 4	

### Data collection

**Table:**

Bruker APEXII area-detector diffractometer	3108 independent reflections
Radiation source: fine-focus sealed tube	2810 reflections with *I* > 2σ(*I*)
graphite	*R*_int_ = 0.032
ω scans	θ_max_ = 27.6°, θ_min_ = 2.4°
Absorption correction: multi-scan (*SADABS*; Sheldrick, 1997)	*h* = −12→12
*T*_min_ = 0.374, *T*_max_ = 0.790	*k* = −15→14
20173 measured reflections	*l* = −18→18

### Refinement

**Table:**

Refinement on *F*^2^	Primary atom site location: structure-invariant direct methods
Least-squares matrix: full	Secondary atom site location: difference Fourier map
*R*[*F*^2^ > 2σ(*F*^2^)] = 0.019	Hydrogen site location: inferred from neighbouring sites
*wR*(*F*^2^) = 0.047	H atoms treated by a mixture of independent and constrained refinement
*S* = 1.06	*w* = 1/[σ^2^(*F*_o_^2^) + (0.0236*P*)^2^ + 1.0326*P*] where *P* = (*F*_o_^2^ + 2*F*_c_^2^)/3
3108 reflections	(Δ/σ)_max_ = 0.001
211 parameters	Δρ_max_ = 0.84 e Å^−^^3^
12 restraints	Δρ_min_ = −0.91 e Å^−^^3^

### Special details

**Table:**

Geometry. All e.s.d.'s (except the e.s.d. in the dihedral angle between two l.s. planes) are estimated using the full covariance matrix. The cell e.s.d.'s are taken into account individually in the estimation of e.s.d.'s in distances, angles and torsion angles; correlations between e.s.d.'s in cell parameters are only used when they are defined by crystal symmetry. An approximate (isotropic) treatment of cell e.s.d.'s is used for estimating e.s.d.'s involving l.s. planes.
Refinement. Refinement of *F*^2^ against ALL reflections. The weighted *R*-factor *wR* and goodness of fit *S* are based on *F*^2^, conventional *R*-factors *R* are based on *F*, with *F* set to zero for negative *F*^2^. The threshold expression of *F*^2^ > σ(*F*^2^) is used only for calculating *R*-factors(gt) *etc*. and is not relevant to the choice of reflections for refinement. *R*-factors based on *F*^2^ are statistically about twice as large as those based on *F*, and *R*- factors based on ALL data will be even larger.

### Fractional atomic coordinates and isotropic or equivalent isotropic displacement parameters (Å^2^)

**Table:**

	*x*	*y*	*z*	*U*_iso_*/*U*_eq_	
Dy1	0.117659 (15)	−0.013113 (10)	−0.167676 (10)	0.01672 (5)	
N1	0.4246 (4)	−0.1300 (3)	−0.3049 (3)	0.0407 (7)	
H1A	0.393 (5)	−0.098 (3)	−0.263 (3)	0.049*	
H1B	0.378 (5)	−0.095 (3)	−0.368 (3)	0.049*	
O1	0.2048 (2)	−0.21224 (15)	−0.15624 (17)	0.0250 (4)	
O1W	0.1053 (3)	−0.02065 (19)	−0.34320 (18)	0.0317 (5)	
H1WA	0.036 (4)	−0.061 (2)	−0.396 (2)	0.038*	
H1WB	0.106 (4)	0.0401 (18)	−0.375 (2)	0.038*	
O2	−0.1030 (2)	−0.13712 (15)	−0.29291 (17)	0.0232 (4)	
O2W	0.5505 (4)	−0.2809 (3)	−0.0349 (3)	0.0669 (9)	
H2WA	0.489 (5)	−0.291 (5)	−0.009 (3)	0.080*	
H2WB	0.624 (4)	−0.238 (4)	0.016 (3)	0.080*	
O3	−0.1939 (2)	−0.31830 (15)	−0.32907 (16)	0.0219 (4)	
O3W	−0.1188 (4)	−0.1547 (2)	−0.5332 (3)	0.0576 (8)	
H3WA	−0.090 (5)	−0.222 (2)	−0.529 (4)	0.069*	
H3WB	−0.209 (3)	−0.155 (3)	−0.543 (4)	0.069*	
O4	0.1169 (2)	−0.39375 (16)	−0.20338 (18)	0.0278 (5)	
O4W	0.6648 (5)	−0.5050 (3)	−0.0068 (3)	0.0701 (11)	
H4WA	0.694 (6)	−0.530 (4)	0.056 (3)	0.084*	
H4WB	0.596 (5)	−0.456 (4)	−0.020 (4)	0.084*	
O5	0.3400 (3)	−0.01122 (16)	0.02214 (18)	0.0263 (5)	
O6	0.0192 (3)	−0.11779 (16)	−0.06741 (17)	0.0276 (4)	
O7	0.0381 (3)	0.11134 (17)	−0.06433 (18)	0.0300 (5)	
O8	0.3894 (3)	−0.01101 (15)	−0.14319 (17)	0.0226 (4)	
C1	0.0965 (3)	−0.2872 (2)	−0.2069 (2)	0.0197 (5)	
C2	−0.0829 (3)	−0.2435 (2)	−0.2832 (2)	0.0180 (5)	
C3	0.4856 (4)	−0.0001 (2)	0.0483 (2)	0.0198 (6)	
C4	−0.0055 (3)	−0.0664 (2)	−0.0010 (2)	0.0220 (6)	
C5	0.3567 (6)	−0.2468 (4)	−0.3333 (4)	0.0761 (15)	
H5A	0.2387	−0.2431	−0.3721	0.091*	
H5B	0.3984	−0.2910	−0.2667	0.091*	
H5C	0.3882	−0.2827	−0.3796	0.091*	
C6	0.6030 (5)	−0.1251 (5)	−0.2451 (4)	0.0710 (14)	
H6A	0.6384	−0.0464	−0.2279	0.085*	
H6B	0.6396	−0.1572	−0.2901	0.085*	
H6C	0.6490	−0.1686	−0.1777	0.085*	

### Atomic displacement parameters (Å^2^)

**Table:**

	*U*^11^	*U*^22^	*U*^33^	*U*^12^	*U*^13^	*U*^23^
Dy1	0.01578 (8)	0.01427 (7)	0.01843 (8)	−0.00014 (4)	0.00807 (6)	−0.00013 (4)
N1	0.0347 (18)	0.0526 (18)	0.0363 (17)	0.0048 (14)	0.0200 (15)	−0.0078 (14)
O1	0.0179 (11)	0.0192 (9)	0.0295 (11)	−0.0011 (8)	0.0073 (9)	−0.0039 (8)
O1W	0.0342 (14)	0.0367 (12)	0.0218 (11)	−0.0059 (10)	0.0133 (11)	−0.0019 (9)
O2	0.0213 (11)	0.0158 (9)	0.0259 (10)	0.0016 (7)	0.0082 (9)	0.0007 (8)
O2W	0.053 (2)	0.0513 (18)	0.066 (2)	0.0025 (14)	0.0119 (17)	−0.0131 (15)
O3	0.0172 (10)	0.0179 (9)	0.0272 (11)	−0.0014 (7)	0.0096 (9)	−0.0029 (7)
O3W	0.066 (2)	0.0331 (13)	0.0650 (19)	0.0039 (13)	0.0292 (19)	0.0093 (13)
O4	0.0228 (11)	0.0161 (9)	0.0319 (11)	0.0016 (8)	0.0063 (9)	−0.0012 (8)
O4W	0.074 (3)	0.068 (2)	0.0376 (18)	0.0164 (15)	0.0098 (18)	−0.0028 (14)
O5	0.0184 (11)	0.0392 (11)	0.0218 (11)	−0.0021 (8)	0.0111 (9)	−0.0007 (8)
O6	0.0340 (12)	0.0239 (10)	0.0308 (12)	−0.0039 (8)	0.0213 (10)	−0.0040 (8)
O7	0.0389 (13)	0.0241 (10)	0.0397 (13)	0.0032 (9)	0.0294 (11)	0.0059 (9)
O8	0.0177 (11)	0.0297 (10)	0.0187 (10)	−0.0005 (7)	0.0085 (9)	−0.0015 (7)
C1	0.0209 (15)	0.0194 (12)	0.0179 (13)	0.0005 (10)	0.0098 (12)	0.0006 (10)
C2	0.0197 (15)	0.0195 (12)	0.0165 (13)	−0.0014 (10)	0.0108 (12)	−0.0012 (10)
C3	0.0205 (15)	0.0161 (11)	0.0200 (14)	0.0015 (10)	0.0090 (12)	0.0005 (9)
C4	0.0157 (14)	0.0231 (14)	0.0252 (15)	−0.0009 (10)	0.0098 (12)	0.0000 (10)
C5	0.096 (4)	0.059 (3)	0.082 (4)	−0.017 (3)	0.054 (3)	−0.020 (2)
C6	0.035 (2)	0.121 (4)	0.052 (3)	0.007 (2)	0.020 (2)	−0.019 (3)

### Geometric parameters (Å, °)

**Table:**

Dy1—O3^i^	2.3846 (17)	O3W—H3WA	0.83 (3)
Dy1—O2	2.3883 (19)	O3W—H3WB	0.80 (4)
Dy1—O5	2.390 (2)	O4—C1	1.249 (3)
Dy1—O6	2.427 (2)	O4—Dy1^ii^	2.4386 (19)
Dy1—O1	2.4335 (18)	O4W—H4WA	0.84 (3)
Dy1—O4^i^	2.4386 (19)	O4W—H4WB	0.82 (4)
Dy1—O8	2.445 (2)	O5—C3	1.248 (4)
Dy1—O1W	2.451 (2)	O6—C4	1.248 (3)
Dy1—O7	2.464 (2)	O7—C4^iii^	1.248 (3)
N1—C6	1.451 (5)	O8—C3^iv^	1.246 (4)
N1—C5	1.463 (6)	C1—C2	1.551 (4)
N1—H1A	0.89 (4)	C3—O8^iv^	1.246 (4)
N1—H1B	0.87 (4)	C3—C3^iv^	1.549 (6)
O1—C1	1.248 (3)	C4—O7^iii^	1.248 (3)
O1W—H1WA	0.84 (4)	C4—C4^iii^	1.544 (5)
O1W—H1WB	0.84 (4)	C5—H5A	0.9600
O2—C2	1.245 (3)	C5—H5B	0.9600
O2W—H2WA	0.86 (5)	C5—H5C	0.9600
O2W—H2WB	0.85 (4)	C6—H6A	0.9600
O3—C2	1.254 (3)	C6—H6B	0.9600
O3—Dy1^ii^	2.3846 (17)	C6—H6C	0.9600
			
O3^i^—Dy1—O2	135.67 (6)	C5—N1—H1B	104 (3)
O3^i^—Dy1—O5	85.20 (6)	H1A—N1—H1B	110 (4)
O2—Dy1—O5	138.66 (7)	C1—O1—Dy1	118.16 (17)
O3^i^—Dy1—O6	135.05 (6)	Dy1—O1W—H1WA	121 (2)
O2—Dy1—O6	70.73 (7)	Dy1—O1W—H1WB	121 (2)
O5—Dy1—O6	74.25 (7)	H1WA—O1W—H1WB	103 (2)
O3^i^—Dy1—O1	143.39 (7)	C2—O2—Dy1	119.42 (17)
O2—Dy1—O1	67.30 (6)	H2WA—O2W—H2WB	99 (2)
O5—Dy1—O1	82.32 (7)	C2—O3—Dy1^ii^	118.80 (17)
O6—Dy1—O1	73.44 (7)	H3WA—O3W—H3WB	107 (3)
O3^i^—Dy1—O4^i^	67.45 (6)	C1—O4—Dy1^ii^	118.02 (17)
O2—Dy1—O4^i^	71.67 (6)	H4WA—O4W—H4WB	105 (3)
O5—Dy1—O4^i^	139.00 (7)	C3—O5—Dy1	121.19 (19)
O6—Dy1—O4^i^	103.57 (7)	C4—O6—Dy1	120.35 (17)
O1—Dy1—O4^i^	137.38 (6)	C4^iii^—O7—Dy1	119.20 (17)
O3^i^—Dy1—O8	71.14 (6)	C3^iv^—O8—Dy1	119.36 (19)
O2—Dy1—O8	124.42 (7)	O1—C1—O4	127.0 (3)
O5—Dy1—O8	66.48 (7)	O1—C1—C2	116.6 (2)
O6—Dy1—O8	130.33 (7)	O4—C1—C2	116.4 (2)
O1—Dy1—O8	72.31 (6)	O2—C2—O3	126.2 (3)
O4^i^—Dy1—O8	125.97 (7)	O2—C2—C1	116.7 (2)
O3^i^—Dy1—O1W	81.96 (7)	O3—C2—C1	117.1 (2)
O2—Dy1—O1W	71.05 (7)	O8^iv^—C3—O5	127.6 (3)
O5—Dy1—O1W	133.30 (8)	O8^iv^—C3—C3^iv^	116.1 (3)
O6—Dy1—O1W	140.18 (7)	O5—C3—C3^iv^	116.3 (3)
O1—Dy1—O1W	81.98 (7)	O7^iii^—C4—O6	126.7 (3)
O4^i^—Dy1—O1W	74.28 (8)	O7^iii^—C4—C4^iii^	116.5 (3)
O8—Dy1—O1W	66.87 (7)	O6—C4—C4^iii^	116.8 (3)
O3^i^—Dy1—O7	69.77 (6)	N1—C5—H5A	109.5
O2—Dy1—O7	111.44 (7)	N1—C5—H5B	109.5
O5—Dy1—O7	71.94 (7)	H5A—C5—H5B	109.5
O6—Dy1—O7	65.99 (7)	N1—C5—H5C	109.5
O1—Dy1—O7	136.30 (7)	H5A—C5—H5C	109.5
O4^i^—Dy1—O7	70.20 (7)	H5B—C5—H5C	109.5
O8—Dy1—O7	124.13 (7)	N1—C6—H6A	109.5
O1W—Dy1—O7	140.87 (7)	N1—C6—H6B	109.5
C6—N1—C5	114.3 (4)	H6A—C6—H6B	109.5
C6—N1—H1A	108 (2)	N1—C6—H6C	109.5
C5—N1—H1A	108 (2)	H6A—C6—H6C	109.5
C6—N1—H1B	112 (3)	H6B—C6—H6C	109.5
			
O3^i^—Dy1—O1—C1	−146.82 (18)	O3^i^—Dy1—O7—C4^iii^	−163.0 (2)
O2—Dy1—O1—C1	−9.33 (19)	O2—Dy1—O7—C4^iii^	64.6 (2)
O5—Dy1—O1—C1	142.0 (2)	O5—Dy1—O7—C4^iii^	−71.4 (2)
O6—Dy1—O1—C1	66.3 (2)	O6—Dy1—O7—C4^iii^	8.9 (2)
O4^i^—Dy1—O1—C1	−25.9 (2)	O1—Dy1—O7—C4^iii^	−14.4 (3)
O8—Dy1—O1—C1	−150.3 (2)	O4^i^—Dy1—O7—C4^iii^	124.6 (2)
O1W—Dy1—O1—C1	−82.1 (2)	O8—Dy1—O7—C4^iii^	−114.7 (2)
O7—Dy1—O1—C1	88.5 (2)	O1W—Dy1—O7—C4^iii^	150.63 (19)
O3^i^—Dy1—O2—C2	156.75 (18)	O3^i^—Dy1—O8—C3^iv^	86.85 (17)
O5—Dy1—O2—C2	−34.0 (2)	O2—Dy1—O8—C3^iv^	−140.12 (16)
O6—Dy1—O2—C2	−67.6 (2)	O5—Dy1—O8—C3^iv^	−6.14 (16)
O1—Dy1—O2—C2	11.96 (19)	O6—Dy1—O8—C3^iv^	−46.9 (2)
O4^i^—Dy1—O2—C2	−179.8 (2)	O1—Dy1—O8—C3^iv^	−95.32 (18)
O8—Dy1—O2—C2	58.7 (2)	O4^i^—Dy1—O8—C3^iv^	128.38 (17)
O1W—Dy1—O2—C2	101.0 (2)	O1W—Dy1—O8—C3^iv^	176.00 (19)
O7—Dy1—O2—C2	−120.7 (2)	O7—Dy1—O8—C3^iv^	39.15 (19)
O3^i^—Dy1—O5—C3	−65.25 (18)	Dy1—O1—C1—O4	−173.8 (2)
O2—Dy1—O5—C3	122.27 (18)	Dy1—O1—C1—C2	6.6 (3)
O6—Dy1—O5—C3	155.14 (19)	Dy1^ii^—O4—C1—O1	−171.9 (2)
O1—Dy1—O5—C3	80.26 (19)	Dy1^ii^—O4—C1—C2	7.7 (3)
O4^i^—Dy1—O5—C3	−112.15 (19)	Dy1—O2—C2—O3	166.6 (2)
O8—Dy1—O5—C3	6.26 (17)	Dy1—O2—C2—C1	−13.2 (3)
O1W—Dy1—O5—C3	9.0 (2)	Dy1^ii^—O3—C2—O2	165.2 (2)
O7—Dy1—O5—C3	−135.52 (19)	Dy1^ii^—O3—C2—C1	−15.0 (3)
O3^i^—Dy1—O6—C4	1.9 (3)	O1—C1—C2—O2	4.2 (4)
O2—Dy1—O6—C4	−134.4 (2)	O4—C1—C2—O2	−175.4 (2)
O5—Dy1—O6—C4	68.0 (2)	O1—C1—C2—O3	−175.6 (2)
O1—Dy1—O6—C4	154.5 (2)	O4—C1—C2—O3	4.7 (4)
O4^i^—Dy1—O6—C4	−69.7 (2)	Dy1—O5—C3—O8^iv^	174.11 (19)
O8—Dy1—O6—C4	106.4 (2)	Dy1—O5—C3—C3^iv^	−5.9 (3)
O1W—Dy1—O6—C4	−151.28 (19)	Dy1—O6—C4—O7^iii^	−171.5 (2)
O7—Dy1—O6—C4	−8.9 (2)	Dy1—O6—C4—C4^iii^	8.4 (4)

Symmetry codes: (i) −*x*, *y*+1/2, −*z*−1/2; (ii) −*x*, *y*−1/2, −*z*−1/2; (iii) −*x*, −*y*, −*z*; (iv) −*x*+1, −*y*, −*z*.

### Hydrogen-bond geometry (Å, °)

**Table:**

*D*—H···*A*	*D*—H	H···*A*	*D*···*A*	*D*—H···*A*
N1—H1A···O8	0.89 (4)	2.00 (4)	2.866 (4)	163 (4)
N1—H1A···O1W	0.89 (4)	2.52 (4)	3.090 (4)	122 (3)
O1W—H1WA···O3W	0.84 (4)	2.03 (2)	2.857 (4)	173 (3)
O4W—H4WB···O2W	0.82 (4)	2.06 (3)	2.767 (4)	144 (5)
N1—H1B···O4W^v^	0.87 (4)	1.91 (4)	2.759 (5)	165 (4)
O1W—H1WB···O3W^vi^	0.84 (4)	1.92 (2)	2.744 (3)	167 (3)
O2W—H2WB···O3^vii^	0.85 (4)	2.06 (2)	2.876 (4)	161 (5)
O3W—H3WA···O7^ii^	0.85 (4)	2.24 (3)	2.959 (3)	145 (4)
O3W—H3WA···O6^viii^	0.83 (4)	2.34 (2)	3.110 (3)	156 (4)
O3W—H3WB···O4W^ix^	0.80 (5)	2.42 (3)	2.959 (5)	125 (4)
O3W—H3WB···O2W^ix^	0.80 (5)	2.49 (2)	3.256 (5)	160 (4)
O4W—H4WA···O4^x^	0.84 (4)	2.10 (3)	2.837 (4)	147 (5)

Symmetry codes: (v) −*x*+1, *y*+1/2, −*z*−1/2; (vi) −*x*, −*y*, −*z*−1; (vii) *x*+1, −*y*−1/2, *z*+1/2; (ii) −*x*, *y*−1/2, −*z*−1/2; (viii) *x*, −*y*−1/2, *z*−1/2; (ix) *x*−1, −*y*−1/2, *z*−1/2; (x) −*x*+1, −*y*−1, −*z*.
